# New-Onset Hidradenitis Suppurativa in Psoriasis Patients: A Multi-Center, Retrospective Cohort Study

**DOI:** 10.3390/life14060730

**Published:** 2024-06-06

**Authors:** Chen-Pi Li, Shao-Wei Lo, Ru-Yin Tsai, Hui-Chin Chang, Shuo-Yan Gau

**Affiliations:** 1 Department of Nursing, Tungs’ Taichung MetroHarbor Hospital, Taichung 435403, Taiwan; g971107@yahoo.com.tw; 2 Education Center, National Cheng Kung University Hospital, College of Medicine, National Cheng Kung University, Tainan 701, Taiwan; c123d456qqq3@gmail.com; 3 Department of Anatomy, School of Medicine, Chung Shan Medical University, Taichung 40201, Taiwan; iris8084@gmail.com; 4 Department of Medical Education, Chung Shan Medical University Hospital, Taichung 40201, Taiwan; 5 Evidence-Based Medicine Center, Chung Shan Medical University Hospital, Taichung 40201, Taiwan; 6 Library, Chung Shan Medical University Hospital, Taichung 40201, Taiwan; 7 School of Medicine, Chung Shan Medical University, Taichung 40201, Taiwan; sixsamurai.shien15@gmail.com; 8 Department of Medical Education, Kaohsiung Chang Gung Memorial Hospital, Kaohsiung 833, Taiwan; 9 Orthopedics Department, Chi-Mei Medical Center, Tainan 71004, Taiwan

**Keywords:** psoriasis, cohort, epidemiology, electronic medical records, hidradenitis suppurativa

## Abstract

Background: Previous research has indicated a potential correlation between hidradenitis suppurativa (HS) and psoriasis (PSO), two chronic inflammatory dermatological diseases. However, there is a lack of comprehensive evaluations that consider a variety of clinical and demographic factors, and the risk of developing HS in PSO patients remains unclear. Our study aims to examine HS risk over time among PSO patients versus matched controls while considering the influence of confounders to provide insights into the potential link between these two diseases. Method: In this multi-institutional cohort study using the TriNetX database, we matched 202,318 patients with PSO with an equivalent number of individuals without PSO, using propensity score matching. The study period extended from 1 January 2005 to 31 December 2018. We computed hazard ratios and their respective 95% confidence intervals (CIs) to evaluate the probability of HS manifestation over a period of 5 years in patients with PSO in comparison to those without PSO. Results: PSO patients demonstrated a consistently higher risk of developing HS than matched controls across all analytic models with the hazard ratios (HR) ranging from 1.43 (95% CI 1.30–1.56) to 5.91 (95% CI 2.49–14.04). Stratified analyses showed the increased HS risk was observed in both genders but only significant in those aged 18–64 years. Kaplan–Meier analysis indicated PSO patients had a higher cumulative probability of developing HS over time (HR 1.77, 95% CI 1.49–1.89). Conclusions: PSO was associated with increased HS risk, highlighting the importance of considering HS as a potential comorbidity in PSO patients and may have implications for early detection, prevention, and management strategies for both conditions. Shared inflammatory pathways, genetic components, and skin dysbiosis may contribute. Further research should elucidate underlying mechanisms.

## 1. Introduction

Psoriasis (PSO) is an inflammatory chronic disease primarily affecting the skin and joints [1]. The prevalence varies widely between countries, ranging from 0.09% to 11.4% [1,2]. Among various morphological and topographical features observed in the PSO, plaque PSO is the most common type, comprising almost 80% of all cases [3]. It appears as distinct, erythematous, scaly plaques frequently coated with silvery scales [3]. The extensor surfaces are the main areas affected by these lesions [3]. PSO was formerly believed to be limited to skin conditions; however, due to its association with several other conditions, including obesity, non-alcoholic fatty liver disease, spondylarthritis, inflammatory bowel diseases (IBDs), uveitis, and cardiovascular events, it is now considered as a systemic inflammatory disease [4,5].

Hidradenitis suppurativa (HS) is an additional persistent skin condition characterized by the inflammation of hair follicles in areas rich in apocrine glands, such as the underarm, groin, and anogenital regions [6,7]. It may eventually result in the formation of abscesses, sinus tracts, and scars [8]. While its prevalence estimates range from 0.7% to 1.2%, with a higher predisposition among females [8], HS persists as a significant medical concern due to its substantial impact on patients’ quality of life and the considerable healthcare costs associated with its management [9]. HS comorbidities have been documented to affect multiple organ systems [10,11,12,13]. Although the precise pathogenesis of HS remains unknown, there is a consensus that it is a multifactorial disease involving immunological factors and the recruitment of self-perpetuating inflammatory mediators [14].

Recent studies have investigated the relationship between PSO and HS. A recent systematic review and meta-analysis by Gau et al. (2022) found that patients with HS had an increased risk of developing PSO [15]. In parallel, a population-based study conducted in Israel by Kridin et al. (2023) revealed an 80% increase in the odds of HS among individuals with PSO [16]. Despite these findings, whether PSO patients have a higher risk of developing HS remains unclear, and the risk of incident HS in patients with PSO has not been comprehensively evaluated in a large-scale, longitudinal study that considers a variety of confounding factors such as clinical, lifestyle, and demographic variables. Furthermore, in the current guidelines for screening for PSO comorbidities, evidence regarding the association between PSO and HS remained insufficient [17].

Therefore, our study aimed to examine the risk of developing HS in a large national cohort of PSO patients by comparing it with matched controls. By filling this research gap, we sought to offer significant insights into the potential link between PSO and HS, which might have implications for early identification, prevention, and management approaches to both diseases.

## 2. Methods and Materials

### 2.1. Data Source, Population Selection and Outcome Measurement

The TriNetX research platform (TriNetX, LLC, Cambridge, MA, USA) was utilized as the primary data source in this multi-institutional cohort investigation. This research platform is a comprehensive global database that amalgamates electronic health records from over 120 participating healthcare entities across the globe. TriNetX has been a cornerstone in prior studies for discerning relationships between various exposures and outcomes [18,19]. The focal point of this study was the US collaborative network, a sub-dataset in the TriNetX research network that primarily comprises records from healthcare organizations within the United States. Our analytical process involved the compilation of data from over 60 healthcare entities nationwide, culminating in approximately 120 million patient records for subsequent examination. Administrative codes, including International Classification of Diseases, Tenth Revision, Clinical Modification (ICD-10-CM) for diseases and RxNorm for medications, were employed to represent exposures, outcomes, and covariates, and are accessible within the TriNetX research network (Table S1).

We selected individuals who had made at least two visits to healthcare facilities and were diagnosed with PSO. The time frame for this selection was between 1 January 2005, and 31 December 2018. The index date was identified as the day when these individuals received their first psoriasis diagnosis. To maintain the integrity and validity of our study population, we implemented several exclusion criteria. We excluded individuals who were younger than 18 years of age, as the focus of our study was on adult populations. We also excluded those individuals who had passed away either before or after the index date. Additionally, individuals with a history of cancer prior to the index date were also excluded from our study. This was done to ensure that our study population was as homogeneous as possible, thereby addressing potential confounding factors. Our study employed a stringent 1:1 propensity score matching (PSM) method. This statistical matching technique allowed us to pair participants based on several vital covariates, thereby reducing the impact of confounding variables on our study outcomes. These covariates encompassed a wide range of factors including age at index, gender, ethnicity, body mass index (BMI), and the existence of comorbidities such as diabetes mellitus, hypertension, hyperlipidemia, Crohn’s disease, and ulcerative colitis. We also considered lifestyle factors such as smoking and alcohol consumption status, and substance-use disorders. Patterns of medical utilization, socioeconomic status, and inflammation-related lab values, such as C-reactive protein levels, were also included in our matching covariates. The PSM process was diligently executed for each analysis. The specific matching covariates were explicitly detailed in the respective table legends to provide transparency and allow for reproducibility of our study methods. Our primary outcome of interest was the occurrence of HS. This was evaluated from the index date to three months after the index date. We instituted this exclusion period to mitigate the potential for reverse causation bias, a common issue in retrospective studies.

### 2.2. Stratification and Sensitivity Analyses

In order to delve into potential variances in the link between PSO and HS across diverse subgroups, we utilized stratification by age and gender. This stratification enabled us to probe whether age or gender had any bearing on the intensity of the association observed. In addition, to tackle potential misclassification bias that is inherent in defining PSO, we carried out sensitivity analyses using various claim-based algorithms. The goal of these analyses was to validate the robustness of our findings by evaluating whether alterations in the definition of PSO had any effect on the observed link with HS. Moreover, given the fact that several systemic drugs for PSO can also play a potential role in incident HS, we also performed analyses including only PSO patients receiving systemic treatments that could potentially influence the incident outcome events (including vitamin D, systemic corticosteroids and TNF alpha inhibitors). We performed sensitivity analyses using a variety of PSM algorithms and wash-out periods. These analyses were pivotal in addressing potential biases, such as over-matching and reverse causation. By employing different PSM algorithms, we sought to evaluate the robustness of the link between PSO and HS under different matching conditions. Likewise, by using different wash-out periods, we were able to examine whether the observed link persisted after excluding incident HS cases that occurred within specific time frames post the index date.

### 2.3. Statistical Analysis

The analyses were carried out within the framework of the TriNetX research network system Analytics function. Each assessment entailed the calculation of hazard ratios (HR) along with their corresponding 95% confidence intervals (95% CI). A systematic application of PSM was employed using a greedy nearest neighbor matching method, with a designated caliper set at 0.1. To evaluate the similarity of baseline patient characteristics across groups, computations of Standardized Mean Difference (SMD) were conducted. Any occurrence where the SMD value surpassed 0.1 signified a considerable and statistically significant disparity between the groups being compared.

### 2.4. Statement of Ethics

This study received approval and was exempted from IRB oversight by the Institutional Review Board of Tungs’ Taichung MetroHarbor Hospital (IRB TTMHH No.:112208N). The research conducted in this study exclusively involved de-identified data from the TriNetX research network, in line with the privacy standards stipulated in the HIPAA Privacy Rule (Section §164.514(a)) [20]. A certified expert, as defined in Section §164.514(b)(1) of the HIPAA Privacy Rule, confirmed the de-identification process of the dataset, which was last updated in December 2020 [20]. This formal confirmation eliminated the requirement for prior IRB exemption from the Western Institutional Review Board (IRB). As a result, research projects that utilize the TriNetX research network are exempt from IRB approval. Compliance with the Strengthening the Reporting of Observational Studies in Epidemiology (STROBE) guideline and the TriNetX publication guideline was maintained throughout this study.

## 3. Results

### 3.1. Baseline Characteristics of the Study Participants

**Figure 1:**
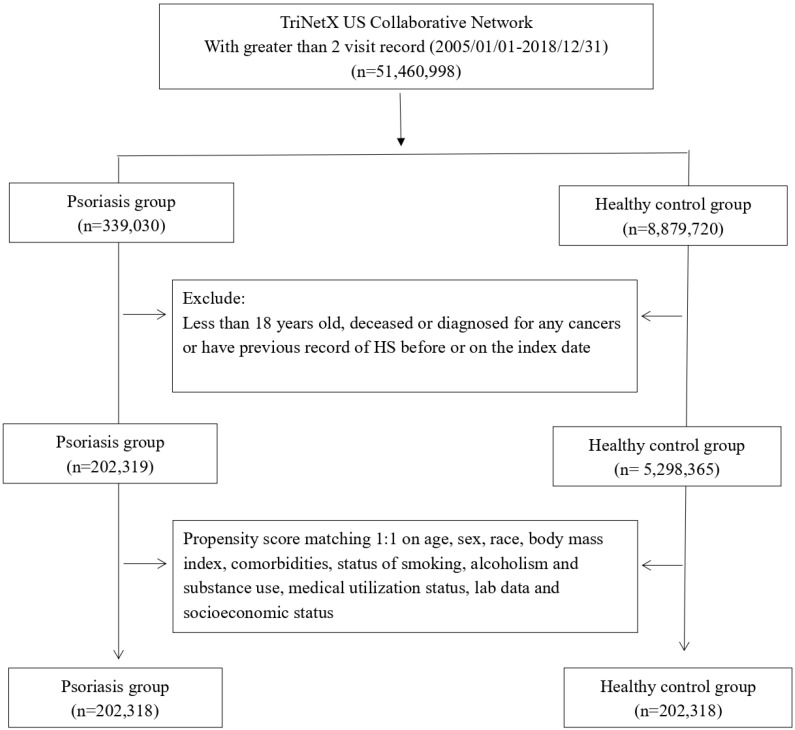
Patient selection process.

In this study, 202,318 patients with PSO and an equivalent number of matched control participants were enrolled in a balanced cohort (Figure 1). Table 1 displays the baseline characteristics of the PSO cohort and the control cohort both before and after propensity score matching.

The PSO cohort’s mean age before matching was 46.1 ± 17.8 years, considerably higher than the control cohort’s mean age of 37.3 ± 20.4 years. There was a significant disparity in the racial composition of the two cohorts: 72.4% of the PSO cohort and 61.6% of the control cohort were White (SMD = 0.23); the proportion of Black or African American individuals was 5.5% in the PSO cohort and 15.4% in the control cohort (SMD = 0.33). Lifestyle factors such as alcohol dependence, smoking, and substance use were more prevalent in the PSO cohort before matching (6.0% vs. 3.6%, SMD = 0.12). Additionally, laboratory data showed a higher percentage of individuals with a CRP ≥ 10 (mg/L) in the PSO cohort (2.3% vs. 0.9%, SMD = 0.12)

After 1:1 propensity matching, the standardized differences were negligible (<0.1) for all matched variables, including age, sex, race, socioeconomic status, lifestyle factors, comorbidities, medical use status, and laboratory data. The mean age of the matched participants was 46.1 ± 17.8 years for the PSO cohort and 46.2 ± 17.8 years for the control cohort. The gender distribution was nearly equal, with women comprising 51.9% of the PSO cohort and 51.8% of the control cohort. In terms of racial demographics, 72.4% of participants in both cohorts identified as White.

### 3.2. Risk of Developing HS

The HR for developing HS was significantly higher in patients with PSO than in non-PSO controls across various models (Table 2).

In the analysis of variated matching covariates, patients with PSO had an increased risk of HS with an HR of 1.425 (95% CI: 1.302, 1.560) in Model 1a, which did not perform propensity score matching. The risk was further elevated in Model 2b, which included age, sex, and race as covariates, with an HR of 1.827 (95% CI: 1.577, 2.117). Model 3c, which further included comorbidities as covariates, showed an HR of 1.715 (95% CI: 1.487, 1.978).

Considering various wash-out periods, the HR for patients with PSO was 1.719 (95% CI: 1.474, 2.005) for 12 months in Model 1d, 1.718 (95% CI: 1.462, 2.02) for 24 months in Model 2e, and 1.825 (95% CI: 1.511, 2.204) for 36 months in Model 3f (Table 3).

With different follow-up times, the HR for patients with PSO was 1.606 (95% CI: 1.433, 1.800) for an 8-year follow-up in Model 1g, 1.637 (95% CI: 1.469, 1.824) for a 10-year follow-up in Model 2h, and 1.637 (95% CI: 1.476, 1.817) for a 15-year follow-up in Model 3i.

When different claim-based algorithms were applied, the HR for patients with PSO was markedly higher. In Model 1j, which included patients with more than two inpatient visits due to PSO, the HR was 2.867 (95% CI: 1.993, 4.125). Model 2k, which included patients diagnosed with PSO with more than two visit records and a prescription of vitamin D, showed an HR of 5.914 (95% CI: 2.492, 14.035). Model 3l, which included patients with more than two visit records and prescribed topical corticosteroids, had an HR of 2.043 (95% CI: 1.746, 2.391). In Models 3m (PSO patients prescribed systemic corticosteroids) and 3n (PSO patients prescribed TNF alpha inhibitors), the risk of HS was 2.497 (95% CI: 2.119, 2.942) and 4.590 (95% CI: 3.072, 6.859), respectively.

**Figure 2:**
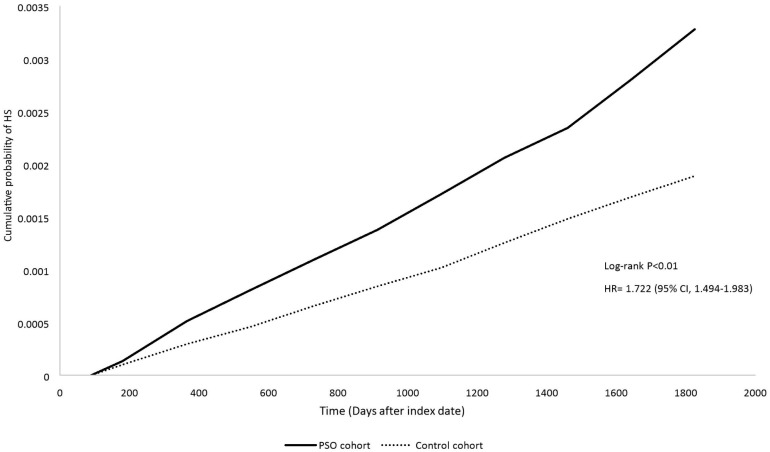
Kaplan–Meier plot in 5-year follow-up. Legends: Matched covariates of propensity matching include age at index, sex, race, body mass index, status of comorbidities (including diabetes mellitus, hypertension, hyperlipidemia, Crohn’s disease, Ulcerative colitis), status of smoking, alcoholism and substance use (mental and behavioral disorders due to psychoactive substance use), medical utilization status and socioeconomic status (problems related to housing and economic circumstances, persons with potential health hazards related to socioeconomic and psychosocial circumstances), and inflammation-associated lab data (C-reacting protein). Wash-out period was set as 3 months after index date.

The Kaplan–Meier plot (Figure 2) demonstrated that the PSO cohort exhibited a significantly higher cumulative probability of HS throughout the 5-year follow-up duration with an HR of 1.772 (95% CI: 1.494, 1.893; log-rank *p* < 0.01).

### 3.3. Stratification Analysis

We performed stratified analyses by gender and age at the index date. The incidence of new-onset HS was higher in the PSO cohort than in the control cohort across both genders. In males, 118 out of 84,286 (0.14%) developed HS, compared with 73 out of 91,250 (0.08%) in the control cohort, with an HR of 1.638 (95% CI: 1.224, 2.193). Similarly, in females, 347 out of 102,059 (0.34%) developed HS in the PSO cohort, whereas 218 out of 103,810 (0.21%) developed HS in the control cohort, with an HR of 1.630 (95% CI: 1.376, 1.931).

When stratified by age, the incidence of HS was significantly higher in the PSO cohort among individuals aged 18–64 years, with 444 cases out of 126,857 (0.35%), as opposed to the control cohort, which had 285 cases out of 129,545 (0.22%), with an HR of 1.582 (95% CI: 1.364, 1.836). Conversely, for individuals aged 65 years, the incidence rates between the PSO and control cohorts did not significantly differ, with an HR of 1.315 (95% CI: 0.869, 1.989).

## 4. Discussion

PSO and HS are persistent inflammatory conditions known for their significant comorbidity load and detrimental effects on health-related quality of life [8,21,22]. To the best of our knowledge, our study is the first large-scale, longitudinal study that assesses the risk of developing HS in PSO patients while considering an extensive array of clinical, lifestyle, and demographic factors. Utilizing the US collaborative network within TriNetX presents a distinct benefit, granting access to an extensive array of patient records specifically from healthcare institutions within the United States. This enables research endeavors that are customized to the demographics, healthcare methodologies, and prevalent conditions within the US healthcare system. Our propensity score-matched study demonstrated a significantly increased risk of developing HS among patients with PSO than among individuals without PSO. The association was consistent across various models after adjusting for potential confounders and sensitivity analyses, suggesting a robust link between these two chronic inflammatory skin conditions.

Common variables and comorbidities, including obesity, smoking, and other components of metabolic syndrome, aggravate both PSO and HS [4,23]. The simultaneous increase in oxidative stress and inflammatory indicators, such as Tumor Necrosis Factor-alpha (TNF-α), is a common characteristic observed in metabolic syndrome. This concurrent elevation is also seen in both PSO and HS. The presence of these elevated markers could potentially trigger these inflammatory skin disorders [24]. To adjust for these confounders, we built Models 1a, 2b, and 3c in Table 2, which varied with the inclusion of matching covariates and comorbidities. Across these models, patients with PSO consistently exhibited a higher HR for developing HS compared with the non-PSO control, strengthening the credibility of the observed association between PSO and new-onset HS, even after adjustment for those confounders. We also conducted sensitivity analyses for different washout periods (Models 1d, 2e, 3f) and follow-up durations (Models 1g, 2h, 3i) to address any misclassification or detection bias. The PSO group had a significantly higher risk of developing HS in all models, which further supports the validity of the observed association. The results of the analysis implied that individuals with PSO exhibited a notably elevated cumulative risk of HS during the entire observation period., which solidifies the temporal relationship between PSO and the subsequent development of HS.

The observed correlation between PSO and an increased risk of developing HS is biologically coherent because both PSO and HS involve immune system dysregulation and share underlying inflammatory pathways. PSO is characterized by chronic inflammation, primarily driven by plasmacytoid dendritic cells (DCs) releasing proinflammatory cytokines such as TNF-α, IFN-α, and IFN-β [25]. These cytokines amplify inflammation by activating inflammatory DCs, which in turn secrete additional TNF-α and IL-23 [25]. IL-23 plays a pivotal role in sustaining the inflammatory response by activating T helper 17 (Th17) and Th22 cells [25]. Persistent inflammation leads to follicular blockage and abnormal immune cell infiltration, which sets the initial stages for HS [26]. The resulting compromised skin barrier and intensified inflammatory environment predispose to the development of HS by allowing bacteria and other pathogens to penetrate hair follicles, culminating in inflammatory nodules and abscesses [27]. HS pathology also involves the IL-23/Th17 axis and the Th1 immune pathway [28,29]. Follicular obstruction stemming from excessive keratinization and epithelial hyperplasia instigates macrophage activation and the release of proinflammatory cytokines, notably IL-1β and TNF [29]. IL-1β facilitates neutrophil migration to affected skin areas, whereas TNF increases the proportion of Th17 cells relative to regulatory T cells, further contributing to immune dysregulation and leukocyte infiltration in HS lesions [29]. Moreover, DCs promote IL-12 and IL-23 production, triggering Th1 and Th17 pathways and perpetuating the inflammatory state [29]. The convergence of cytokines implicated in both conditions rationalizes the efficacy of biological therapies for HS. For example, TNF-α inhibitors, such as adalimumab, and IL-17 inhibitors, like brodalumab, have demonstrated beneficial effects in patients with both HS and PSO by targeting common inflammatory mechanisms [30,31,32].

Genetic factors may play a role in the link between PSO and HS. PSO is a complex genetic disorder, with genetic elements accounting for approximately 70% of the predisposition to the disease [33]. This genetic connection is evidenced by the increased occurrence of PSO among family members globally [33]. Similarly, nearly 30% of individuals with HS report a family history of the condition, and certain familial cases of HS are believed to follow an autosomal dominant pattern of inheritance [34]. Numerous genes have also been implicated in sporadic cases of HS [35]. Although the precise genetic links between PSO and HS are yet to be determined, both PSO and HS are classified as autoinflammatory keratinization diseases, which involve excessive activation of the innate immune system due to genetic predisposing factors, such as IL-36RN and CARD14, as well as components of the IL-1 pathway, such as IL1RN, TNF receptor 1 (TNFRSF1A), and TNFAIP3 [36,37]. Furthermore, “Psoriatic Arthritis, Pyoderma Gangrenosum, Acne, and Suppurative Hidradenitis”, a rare genetic disease also known as PsAPASH syndrome, is thought to arise from mutations in the PSTPIP1 gene. This provides additional support for the theory that genetic factors may underlie the association between PSO and HS. Further research is essential to uncover the exact genetic connections that predispose individuals with PSO to a higher risk of developing HS.

The skin microbiome plays a crucial role in maintaining a balanced immune response and preventing excessive inflammation [38]. PSO results in the disruption of the skin microbiome, promoting the growth of specific bacteria such as *Streptococcus* species and *Staphylococcus aureus* [39]. Through the production of toxins, enzymes, and other chemicals that stimulate the immune system, these bacteria can further activate inflammatory reactions [39]. Furthermore, the imbalance in the skin microbiome may be exacerbated by immunological dysregulation linked to PSO, continuing the cycle of inflammation and dysbiosis [40]. Pathogenic species that can directly damage skin cells and aggravate inflammatory responses may become more prevalent because of the overabundance of pro-inflammatory bacteria and the disappearance of beneficial commensal bacteria [40]. The proliferation of certain bacterial species, such as Staphylococcus aureus, Corynebacterium, and Porphyromonas, can lead to the formation of HS lesions [27]. 

Remarkably, the risk of developing HS was substantially higher when claim-based algorithms were used to identify PSO patients with more specific diagnostic criteria and treatment patterns (models 1j, 2k, 3l). This suggests that patients with more severe or well-established PSO may have a greater risk of developing HS. Patients with more than two inpatient visits due to PSO had an HR of 2.867 (95% CI: 1.993, 4.125). Frequent hospitalizations may be indicative of more severe disease states as they are often accompanied by more severe systemic inflammation, which could predispose patients to developing HS.

Moreover, PSO patients with more than two visit records who received a topical corticosteroid prescription had a roughly two-fold increased risk of developing HS. Topical corticosteroids are anti-inflammatory drugs used to treat PSO [41]. While intralesional corticosteroid is recommended for short-term control of HS flares [42], it is unclear how topical corticosteroids function in HS. Short-term use may help lower inflammation, but prolonged use may change the skin’s immunological response and damage the follicular structure [43], making patients more prone to developing HS.

It is interesting to note that the HR was 5.914 (95% CI: 2.492, 14.035) for PSO patients with more than two visit records who received a vitamin D prescription. Given vitamin D’s reported benefits for both PSO and HS [41], this noticeably elevated risk may appear paradoxical. In patients with PSO, topical vitamin D analogs bind to vitamin D receptors on skin cells, normalizing the growth cycle and reducing the rapid proliferation characteristic of the condition [44]. Vitamin D also possesses immunomodulatory effects, regulating the immune response and reducing inflammation and subsequent plaque formation [44]. While topical vitamin D analogs are beneficial for PSO, oral supplementation at doses that avoid hypercalcemia and calciuria does not have a positive effect on the condition [41]. As a result, it is not recommended for the treatment of PSO [41]. Studies show a higher prevalence of vitamin D deficiency among individuals with HS [45]. In addition, Guillet et al. (2015) found that 79% of people with HS and a vitamin D deficiency responded to supplementation, experiencing at least a 20% decrease in the number of nodules and flare-ups after six months [46]. Diotallevi et al. (2022) revealed that 50% of people with HS experienced a relapse after discontinuing vitamin D supplements [47], suggesting that vitamin D plays a role in preventing flare-ups. Therefore, we proposed that the observed paradox of a high HR in patients who are prescribed vitamin D may imply that the utilization of vitamin D commonly as a substitute or supplementary treatment to corticosteroids is indicative of more severe PSO, which in turn, increase the risk of developing HS. Further research is needed to explore the underlying mechanism behind this observation and the potential interplay between vitamin D, PSO severity, and HS development.

Stratified analyses revealed an increased risk of HS in both male and female patients with PSO, suggesting that the association between PSO and HS is not gender-specific, which is consistent with the results of a previous study by Kridin et al. (2023) [16]. In addition, elevated risk was primarily observed in individuals aged 18–64 years, whereas the risk was not significantly different between PSO and non-PSO cohorts in individuals aged 65 years and above. Consistent with this, Kridin et al. (2023) reported that patients with concomitant PSO and HS were younger (<60 years old) than those with isolated PSO [16]. This age-related finding may be attributed to various factors, including potential differences in disease severity, comorbidities, or treatment patterns between younger and older populations. Both PSO and HS generally appear at earlier ages, with PSO’s age-specific incidence rates displaying a double peak around the ages of 30–39 and a secondary peak around the ages of 50–59 or 60–69 [1,2], and HS having a peak incidence between 20 and 40 years [8]. With increasing age, the immune system undergoes immunosenescence, which is characterized by a gradual decline in immune function and dysregulation of inflammatory responses, potentially mitigating the inflammatory processes and immune dysregulation that may contribute to the development of HS in individuals with PSO [48].

Our study has several strengths. First, we used a large sample size in each cohort, increasing our findings’ statistical power and precision. Second, we used propensity score matching methods that enabled us to obtain similar baseline characteristics between the PSO and control groups and built models to evaluate the influence of diverse variables on risk, significantly mitigating confounding bias. Third, we performed sensitivity analyses and demonstrated the robustness of our findings. However, our study also has some limitations that should be acknowledged. First, we used administrative data, which may have some inaccuracies or incompleteness in diagnosing and coding HS and other variables. Moreover, in the current dataset, we were not able to confirm whether or not the diagnosis of HS and PSO were made by specialists. In this case, potential misclassification bias should be noted. Second, because our study primarily included white people (72.4%), additional research is required to investigate this in patients of diverse ethnic origins. Third, the severity indexes of both diseases, such as the Psoriasis Area and Severity Index (PASI) and Hidradenitis Suppurativa Physician’s Global Assessment (HS-PGA) score, were not available in the TriNetX database. In this case, we were not able to evaluate the influence caused by disease severity on the observed PSO–HS association.

Despite these limitations, our study provides evidence that patients with PSO have a significantly increased risk of developing HS. Given the substantial burden of HS on patients’ quality of life and the associated healthcare costs [9], the identification of PSO as a potential risk factor for HS has important clinical implications. In the current study, by tapping into data from a varied range of healthcare entities, investigators are potentially privy to a plethora of real-world patient data, facilitating comprehensive analyses and insights into a multitude of health conditions and therapeutic interventions. Doctors treating patients with PSO should be cognizant of this heightened risk and contemplate screening for preliminary indications of HS, especially in the high-risk subgroups pinpointed in this research. Prompt detection and treatment of HS could aid in averting disease advancement and diminishing related morbidity, and systemic treatments proven to be effective for both conditions might be favored.

In conclusion, our study provides robust evidence of an increased risk of developing HS in patients with PSO, particularly among younger individuals and those with more severe or well-established PSO. These findings highlight the need for increased awareness, screening, and multidisciplinary management of patients with PSO to address the potential development of comorbid inflammatory conditions such as HS. It is recommended that future research should focus on elucidating the underlying mechanisms and developing preventive and therapeutic strategies tailored to this high-risk population.

## Figures and Tables

**Table 1:** Baseline characteristics.

	Before Matching			After Matching ^a^		
	Psoriasis Cohort (n = 202,319)	Control Cohort (n = 5,298,365)	SMD	Psoriasis Cohort (n = 202,318)	Control Cohort (n = 202,318)	SMD
Age at index						
Mean±SD	46.1 ± 17.8	37.3 ± 20.4	**0.25**	46.1 ± 17.8	46.2 ± 17.8	0.00
Sex						
Male	89,040 (44.0)	2,213,221 (41.8)	0.05	89,040 (44.0)	88,076 (43.5)	0.01
Female	104,961 (51.9)	2,894,881 (54.6)	0.06	104,960 (51.9)	104,776 (51.8)	0.00
Race, n (%)						
White	146,386 (72.4)	3,265,883 (61.6)	**0.23**	146,385 (72.4)	146,468 (72.4)	0.00
Black or African American	11,193 (5.5)	813,195 (15.4)	**0.33**	11,193 (5.5)	11,187 (5.5)	0.00
Asian	6757 (3.3)	191,275 (3.6)	0.01	6757 (3.3)	6934 (3.4)	0.00
American Indian or Alaska Native	621 (0.3)	15,834 (0.3)	0.00	621 (0.3)	556 (0.3)	0.01
Native Hawaiian or Other Pacific Islander	762 (0.4)	11,993 (0.2)	0.03	762 (0.4)	475 (0.2)	0.03
Socioeconomic status						
Socioeconomic/psychosocial circumstances problem	1543 (0.8)	41,806 (0.8)	0.00	1543 (0.8)	1411 (0.7)	0.01
Lifestyle						
Alcohol dependence, smoking, and substance use	12,207 (6.0)	188,301 (3.6)	**0.12**	12,206 (6.0)	12,238 (6.0)	0.00
Comorbidities						
Hypertension	27,234 (13.5)	552,020 (10.4)	0.09	27,233 (13.5)	27,206 (13.4)	0.00
Diabetes mellitus	12,021 (5.9)	222,274 (4.2)	0.08	12,020 (5.9)	11,998 (5.9)	0.00
Hyperlipidemia	17,926 (8.9)	362,929 (6.9)	0.07	17,925 (8.9)	17,884 (8.8)	0.00
Chronic Kidney Disease	2269 (1.1)	47,135 (0.9)	0.02	2269 (1.1)	2319 (1.1)	0.00
Depression	11,861 (5.9)	218,367 (4.1)	0.08	11,861 (5.9)	10,726 (5.3)	0.02
Ulcerative colitis	817 (0.4)	8471 (0.2)	0.05	816 (0.4)	664 (0.3)	0.01
Crohn’s disease	1562 (0.8)	9740 (0.2)	0.09	1561 (0.8)	1383 (0.7)	0.01
Medical Utilization Status						
Ambulatory visit	111,010 (54.9)	2,837,611 (53.6)	0.03	111,009 (54.9)	111,015 (54.9)	0.00
Inpatient visit	21,508 (10.6)	610,379 (11.5)	0.03	21,507 (10.6)	21,414 (10.6)	0.00
Laboratory data						
BMI, n (%)						
≥25 (kg/m^2^)	19,661 (9.7)	409,587 (7.7)	0.07	19,660 (9.7)	19,517 (9.6)	0.00
CRP, n (%)						
≥10 (mg/L)	4726 (2.3)	46,547 (0.9)	**0.12**	4725 (2.3)	4578 (2.3)	0.00

Bold font represents a standardized difference was more than 0.1. PSO, psoriasis; HS: Hidradenitis Suppurativa; SMD, standardized mean difference. ^a^ Matched covariates of propensity matching include age at index, sex, race, body mass index, status of comorbidities (including diabetes mellitus, hypertension, hyperlipidemia, Crohn’s disease, Ulcerative colitis), status of smoking, alcoholism and substance use (mental and behavioral disorders due to psychoactive substance use), medical utilization status and socioeconomic status (problems related to housing and economic circumstances, persons with potential health hazards related to socioeconomic and psychosocial circumstances), and inflammation-associated lab data (C-reacting protein).

**Table 2:** Hazard ratio of hidradenitis suppurativa with 95% confidence interval under various models.

Various matching covariates	Model 1 ^a^	Model 2 ^b^	Model 3 ^c^		
Non-PSO controls	1.00	1.00	1.00		
PSO patients	1.425 (1.302,1.560)	1.827 (1.577,2.117)	1.715 (1.487,1.978)		
Various wash-out periods	Model 1 ^d^	Model 2 ^e^	Model 3 ^f^		
Non-PSO controls	1.00	1.00	1.00		
PSO patients	1.719 (1.474,2.005)	1.718 (1.462,2.02)	1.825 (1.511,2.204)		
Various follow-up times	Model 1 ^g^	Model 2 ^h^	Model 3 ^i^		
Non-PSO controls	1.00	1.00	1.00		
PSO patients	1.606 (1.433,1.800)	1.637 (1.469,1.824)	1.637 (1.476,1.817)		
Various claim-based algorithms	Model 1 ^j^	Model 2 ^k^	Model 3 ^l^	Model 4 ^m^	Model 5 ^n^
Non-PSO controls	1.00	1.00	1.00	1.00	1.00
PSO patients	2.867 (1.993,4.125)	5.914 (2.492,14.035)	2.043 (1.746,2.391)	2.497 (2.119,2.942)	4.590 (3.072,6.859)

PSO, psoriasis; HS, hidradenitis suppurativa. In this table, aside from the analyses of variated matching covariates, propensity score matching was presented in all analyses, with the covariates of age at index, sex, race, body mass index, status of comorbidities (including diabetes mellitus, hypertension, hyperlipidemia, Crohn’s disease, Ulcerative colitis), status of smoking, alcoholism and substance use (mental and behavioral disorders due to psychoactive substance use), medical utilization status and socioeconomic status (problems related to housing and economic circumstances, persons with potential health hazards related to socioeconomic and psychosocial circumstances), and inflammation-associated lab data (C-reacting protein). ^a^ Crude model without performing propensity score matching. ^b^ Covariates of propensity score matching include age at index, sex, and race. ^c^ Covariates of propensity score matching include age at index, sex, race, and comorbidities. ^d^ Wash-out period was set as 12 months in this model. Incident hidradenitis suppurativa that occurred within 12 months were not calculated as outcome events. ^e^ Wash-out period was set as 24 months in this model. Incident hidradenitis suppurativa that occurred within 24 months were not calculated as outcome events. ^f^ Wash-out period was set as 36 months in this model. Incident hidradenitis suppurativa that occurred within 36 months were not calculated as outcome events. ^g^ Follow-up period was set as 8 years in this model. ^h^ Follow-up period was set as 10 years in this model. ^i^ Follow-up period was set as 15 years in this model. ^j^ Only patients with more than 2 inpatient visit records due to psoriasis were included as the psoriasis group in this model. ^k^ Only patients diagnosed with psoriasis with more than 2 visit records and with a prescription of vitamin D were included as the psoriasis group in this model. ^l^ Only patients diagnosed with psoriasis with more than 2 visit records and with a prescription of topical corticosteroids were included as the psoriasis group in this model. ^m^ Only patients diagnosed with psoriasis with more than 2 visit records and with a prescription of systemic corticosteroids were included as the psoriasis group in this model. ^n^ Only patients diagnosed with psoriasis with more than 2 visit records and with a prescription of TNF alpha inhibitors (adalimumab or infliximab) were included as the psoriasis group in this model.

**Table 3:** Stratification analysis of hidradenitis suppurativa risk in psoriasis patients in 5-year follow-up.

	Cases Occurring New-Onset Hidradenitis Suppurativa		
Subgroups	PSO Cohort No. of Outcome Event (%)	Control Cohort No. of Outcome Event (%)	HR (95% CI) ^a^
Gender			
Male	118(0.14)	73(0.08)	1.638 (1.224, 2.193)
Female	347(0.34)	218(0.21)	1.630 (1.376, 1.931)
Age at index date			
18–64 years old	444(0.35)	285(0.22)	1.582 (1.364, 1.836)
≥65 years old	51(0.07)	40(0.06)	1.315 (0.869, 1.989)

^a^ Propensity score matching was presented in all analyses, with the covariates of age at index, sex, race, body mass index, status of comorbidities (including diabetes mellitus, hypertension, hyperlipidemia, Crohn’s disease, Ulcerative colitis), status of smoking, alcoholism and substance use (mental and behavioral disorders due to psychoactive substance use), medical utilization status and socioeconomic status (problems related to housing and economic circumstances, persons with potential health hazards related to socioeconomic and psychosocial circumstances), and inflammation-associated lab data (C-reacting protein).

## Data Availability

Data in this study were retrieved from the TriNetX Research Network. All data available in the database were administrated by the TriNetX platform. Detailed information can be retrieved at the official website of the research network (https://trinetx.com). Upon appropriate requests, the analyzed datasets in this study could be provided by the authors. Datasets analyzed in the present study can be provided by the authors upon reasonable request.

## Supplementary Materials

The following supporting information can be downloaded at: https://www.mdpi.com/article/10.3390/life14060730/s1, Table S1. Utilized proxy codes.

## References

[1] Parisi R., Symmons D.P., Griffiths C.E., Ashcroft D.M. (2013). Global epidemiology of psoriasis: A systematic review of incidence and prevalence. J. Investig. Dermatol..

[2] Griffiths C., Van der Walt J., Ashcroft D., Flohr C., Naldi L., Nijsten T., Augustin M. (2017). The Global State of Psoriasis Disease Epidemiology: A Workshop Report.

[3] Motolese A., Ceccarelli M., Macca L., Li Pomi F., Ingrasciotta Y., Nunnari G., Guarneri C. (2022). Novel therapeutic approaches to psoriasis and risk of infectious disease. Biomedicines.

[4] Borgia F., Ciodaro F., Guarneri F., Bartolotta A., Papaianni V., Guarneri C., Catalano N., Galletti F., Cannavò S.P. (2018). Auditory System Involvement in Psoriasis. Acta Derm. Venereol..

[5] Vata D., Tarcau B.M., Popescu I.A., Halip I.A., Patrascu A.I., Gheuca Solovastru D.F., Mocanu M., Chiriac P.C., Gheuca Solovastru L. (2023). Update on Obesity in Psoriasis Patients. Life.

[6] Li Pomi F., Macca L., Motolese A., Ingrasciotta Y., Berretta M., Guarneri C. (2021). Neoplastic implications in patients suffering from hidradenitis suppurativa under systemic treatments. Biomedicines.

[7] Jemec G.B. (2012). Hidradenitis suppurativa. N. Engl. J. Med..

[8] Nguyen T.V., Damiani G., Orenstein L.A., Hamzavi I., Jemec G. (2021). Hidradenitis suppurativa: An update on epidemiology, phenotypes, diagnosis, pathogenesis, comorbidities and quality of life. J. Eur. Acad. Dermatol. Venereol..

[9] Marvel J., Vlahiotis A., Sainski-Nguyen A., Willson T., Kimball A. (2019). Disease burden and cost of hidradenitis suppurativa: A retrospective examination of US administrative claims data. BMJ Open.

[10] Chang H.-C., Wu C.-L., Chiu T.-M., Liao W.-C., Gau S.-Y. (2023). Risk of osteoarthritis in patients with hidradenitis suppurativa: A global federated health network analysis. Front. Immunol..

[11] Gau S.Y. (2023). Increased risk of renal diseases in people with hidradenitis suppurativa: A systematic review and meta-analysis. Int. J. Dermatol..

[12] Gau S.Y., Chan W.L., Tsai J.D. (2023). Risk of Atopic Diseases in Patients with Hidradenitis Suppurativa: A Systematic Review and Meta-Analysis of Observational Studies. Dermatology.

[13] Li C.P., Chen S.J., Tsai R.Y., Chang H.C., Gau S.Y. (2024). Patients with hidradenitis suppurativa are associated with a high risk of cholecystitis and pancreatitis: A retrospective cohort study. Int. J. Dermatol..

[14] Macca L., Moscatt V., Ceccarelli M., Ingrasciotta Y., Nunnari G., Guarneri C. (2022). Hidradenitis Suppurativa in patients with HIV: A scoping review. Biomedicines.

[15] Gau S.-Y., Preclaro I.A.C., Wei J.C.-C., Lee C.-Y., Kuan Y.-H., Hsiao Y.-P., Juang S.-E., Ma K.S.-K. (2022). Risk of psoriasis in people with hidradenitis suppurativa: A systematic review and meta-analysis. Front. Immunol..

[16] Kridin K., Shani M., Schonmann Y., Fisher S., Shalom G., Comaneshter D., Batat E., Cohen A.D. (2023). Psoriasis and hidradenitis suppurativa: A large-scale population-based study. J. Am. Acad. Dermatol..

[17] Roubille C., Richer V., Starnino T., McCourt C., McFarlane A., Fleming P., Siu S., Kraft J., Lynde C., Pope J. (2015). Evidence-based recommendations for the management of comorbidities in rheumatoid arthritis, psoriasis, and psoriatic arthritis: Expert opinion of the Canadian Dermatology-Rheumatology Comorbidity Initiative. J. Rheumatol..

[18] Gau S.Y., Guo Y.C., Lu H.Y., Lin C.Y., Lee C.Y., Tsai R.Y., Chang H.C., Wu M.C., Li C.P. (2024). Hidradenitis suppurativa as a potential risk factor of periodontitis: A multi-center, propensity-score-matched cohort study. Int. J. Med. Sci..

[19] Gau S.Y., Liu P.Y., Chen S.N., Chiu T.M., Tsai R.Y., Chang H.C., Li C.P. (2024). Risk of Keratitis and Keratopathy in Hidradenitis Suppurativa Patients: A Global Federated Health Network Analysis. In Vivo.

[20] (2000). HIPAA Privacy Rule.

[21] Obradors M., Blanch C., Comellas M., Figueras M., Lizan L. (2016). Health-related quality of life in patients with psoriasis: A systematic review of the European literature. Qual. Life Res..

[22] Di Spirito F., Raimondo A., Di Palo M.P., Martina S., Fordellone M., Rosa D., Amato M., Lembo S. (2024). Oral Lesions and Oral Health-Related Quality of Life in Adult Patients with Psoriasis: A Retrospective Chart Review. Life.

[23] Mohammadi S., Gholami A., Hejrati L., Rohani M., Rafiei-Sefiddashti R., Hejrati A. (2021). Hidradenitis suppurativa; classification, remedies, etiology, and comorbidities; A narrative review. J. Fam. Med. Prim. Care.

[24] Rohm T.V., Meier D.T., Olefsky J.M., Donath M.Y. (2022). Inflammation in obesity, diabetes, and related disorders. Immunity.

[25] Furue K., Ito T., Furue M. (2018). Differential efficacy of biologic treatments targeting the TNF-α/IL-23/IL-17 axis in psoriasis and psoriatic arthritis. Cytokine.

[26] Sabat R., Jemec G.B., Matusiak Ł., Kimball A.B., Prens E., Wolk K. (2020). Hidradenitis suppurativa. Nat. Rev. Dis. Primers.

[27] Wark K.J., Cains G.D. (2021). The microbiome in hidradenitis suppurativa: A review. Dermatol. Ther..

[28] Schlapbach C., Hänni T., Yawalkar N., Hunger R.E. (2011). Expression of the IL-23/Th17 pathway in lesions of hidradenitis suppurativa. J. Am. Acad. Dermatol..

[29] Amat-Samaranch V., Agut-Busquet E., Vilarrasa E., Puig L. (2021). New perspectives on the treatment of hidradenitis suppurativa. Ther. Adv. Chronic Dis..

[30] Pinter A., Sarlak M., Zeiner K.N., Malisiewicz B., Kaufmann R., Romanelli M., Koenig A., Chiricozzi A. (2021). Coprevalence of hidradenitis suppurativa and psoriasis: Detailed demographic, disease severity and comorbidity pattern. Dermatology.

[31] Gkanti V., Dalamaga M., Papadavid E. (2023). Drug survival of brodalumab is greater in patients with psoriasis and psoriatic arthritis in a real-world setting. Int. J. Dermatol..

[32] Kashetsky N., Mufti A., Alabdulrazzaq S., Lytvyn Y., Sachdeva M., Rahat A., Yeung J. (2022). Treatment outcomes of IL-17 inhibitors in hidradenitis suppurativa: A systematic review. J. Cutan. Med. Surg..

[33] Guilhou J.-J., Molès J.-P. (2008). New hypotheses in the genetics of psoriasis and other ‘complex’ diseases. Dermatology.

[34] Ingram J.R. (2016). The genetics of hidradenitis suppurativa. Dermatol. Clin..

[35] Sun Q., Broadaway K.A., Edmiston S.N., Fajgenbaum K., Miller-Fleming T., Westerkam L.L., Melendez-Gonzalez M., Bui H., Blum F.R., Levitt B. (2023). Genetic variants associated with hidradenitis suppurativa. JAMA Dermatol..

[36] Osmola-Mańkowska A., Teresiak-Mikołajczak E., Skrzypczak-Zielińska M., Adamski Z. (2018). Genetic polymorphism in psoriasis and its meaning for the treatment efficacy in the future. Postepy Dermatol. Alergol..

[37] Singh S., Pradhan D., Puri P., Ramesh V., Aggarwal S., Nayek A., Jain A. (2019). Genomic alterations driving psoriasis pathogenesis. Gene.

[38] Balato A., Cacciapuoti S., Di Caprio R., Marasca C., Masarà A., Raimondo A., Fabbrocini G. (2019). Human microbiome: Composition and role in inflammatory skin diseases. Arch. Immunol. Ther. Exp..

[39] Liang X., Ou C., Zhuang J., Li J., Zhang F., Zhong Y., Chen Y. (2021). Interplay between skin microbiota dysbiosis and the host immune system in psoriasis: Potential pathogenesis. Front. Immunol..

[40] Zouboulis C.C., Benhadou F., Byrd A.S., Chandran N.S., Giamarellos-Bourboulis E.J., Fabbrocini G., Frew J.W., Fujita H., González-López M.A., Guillem P. (2020). What causes hidradenitis suppurativa?—15 years after. Exp. Dermatol..

[41] Elmets C.A., Korman N.J., Prater E.F., Wong E.B., Rupani R.N., Kivelevitch D., Armstrong A.W., Connor C., Cordoro K.M., Davis D.M. (2021). Joint AAD–NPF Guidelines of care for the management and treatment of psoriasis with topical therapy and alternative medicine modalities for psoriasis severity measures. J. Am. Acad. Dermatol..

[42] Alikhan A., Sayed C., Alavi A., Alhusayen R., Brassard A., Burkhart C., Crowell K., Eisen D.B., Gottlieb A.B., Hamzavi I. (2019). North American clinical management guidelines for hidradenitis suppurativa: A publication from the United States and Canadian Hidradenitis Suppurativa Foundations: Part I: Diagnosis, evaluation, and the use of complementary and procedural management. J. Am. Acad. Dermatol..

[43] Niculet E., Bobeica C., Tatu A.L. (2020). Glucocorticoid-induced skin atrophy: The old and the new. Clin. Cosmet. Investig. Dermatol..

[44] Brożyna A.A., Slominski R.M., Nedoszytko B., Zmijewski M.A., Slominski A.T. (2022). Vitamin D signaling in psoriasis: Pathogenesis and therapy. Int. J. Mol. Sci..

[45] Seetan K., Eldos B., Saraireh M., Omari R., Rubbai Y., Jayyusi A., Abu Jubran M. (2022). Prevalence of low vitamin D levels in patients with Hidradenitis suppurativa in Jordan: A comparative cross-sectional study. PLoS ONE.

[46] Guillet A., Brocard A., Bach Ngohou K., Graveline N., Leloup A.G., Ali D., Nguyen J.M., Loirat M.J., Chevalier C., Khammari A. (2015). Verneuil’s disease, innate immunity and vitamin D: A pilot study. J. Eur. Acad. Dermatol. Venereol..

[47] Diotallevi F., Campanati A., Martina E., Radi G., Paolinelli M., Marani A., Molinelli E., Candelora M., Taus M., Galeazzi T. (2022). The role of nutrition in immune-mediated, inflammatory skin disease: A narrative review. Nutrients.

[48] Ventura M.T., Casciaro M., Gangemi S., Buquicchio R. (2017). Immunosenescence in aging: Between immune cells depletion and cytokines up-regulation. Clin. Mol. Allergy.

