# Supplementary material for: New-Onset Hidradenitis Suppurativa in Psoriasis Patients: A Multi-Center, Retrospective Cohort Study
Source: Life (Basel). 2024 Jun 6;14(6):730. doi: 10.3390/life14060730 (PMC11205059; doi:10.3390/life14060730)
Supplement: Supplementary file 1 [file life-14-00730-s001.zip › life-3022357-supplementary.pdf]

Supplementary Files

Supplementary Tables

Table S1. Utilized proxy codes [a].

**Description**	**ICD-10-CM codes**
Psoriasis	L40
Hidradenitis Suppurativa	L73.2
Neoplasms	C00-D49
Diabetes mellitus	E08-E13
Hypertension	I10
Hyperlipidemia	E78.5
Socioeconomic and psychosocial circumstances	Z55-Z65
Substance abuse	F10-F19
Depression	F32
Chronic kidney disease	N18
Ulcerative colitis	K51
Crohn's disease	K50
Encounter for general examination	Z00
**Medications**	**ATC codes/RxNorm**
Topical Corticosteroids	ATC code: D07
Vitamin D	RxNorm: 11253
Systemic Corticosteroids	ATC code: H02
Adalimumab	RxNorm: 327361
Infliximab	RxNorm: 191831

[a] ICD-10-CM: International Classification of Diseases, Tenth Revision, Clinical Modification; ATC codes: Anatomical Therapeutic Chemical codes.
